# Effect of Prolonged and Substantial Weight Loss on Incident Atrial Fibrillation: A Systematic Review and Meta-Analysis

**DOI:** 10.3390/nu15040940

**Published:** 2023-02-14

**Authors:** Antonio E. Pontiroli, Lucia Centofanti, Carel W. Le Roux, Silvia Magnani, Elena Tagliabue, Franco Folli

**Affiliations:** 1 Dipartimento di Scienze della Salute, Università degli Studi di Milano, 20133 Milan, Italy; 2 Diabetes Complications Research Centre, University College Dublin, D04 V1W8 Dublin, Ireland; 3 Ospedale San Paolo, 20142 Milan, Italy; 4 IRCCS MultiMedica, Value-Based Healthcare Unit, 20099 Milan, Italy

**Keywords:** surgery, bariatric surgery, atrial fibrillation, weight loss, obesity, meta-analysis, body weight, body mass index

## Abstract

**Background**. Overweight and obesity are associated with atrial fibrillation (AF), and bariatric surgery (BS), able to induce sustained and prolonged weight loss, might represent the ideal treatment in the prevention of AF. Previous studies could not definitely establish a role for weight loss and BS in preventing incident AF so far. During the last few years, several studies on the effect of bariatric surgery on cardiovascular diseases have been published, and we performed a systematic review and meta-analysis to evaluate the role of weight loss through BS in the prevention of incident AF in obesity. **Methods**. This meta-analysis followed the PRISMA guideline. Eligible studies were controlled trials evaluating the appearance of atrial fibrillation in patients undergoing weight loss through BS as compared with patients receiving medical treatment. Quality of studies was assessed according to the Newcastle-Ottawa Quality Assessment Scale, and risk-of-bias was evaluated employing the Egger’s test. All analyses were run by a random-effects model according to Hartung and Knapp and sensitivity analyses were performed. Heterogeneity was assessed through Q and I^2^ statistics for each comparison, and potential publication bias was formally investigated. **Results**. Ten studies were included in the meta-analysis, and the overall result was statistically significant [OR = 0.665 (0.475–0.929), *p* = 0.017], with significant heterogeneity (Q = 48.98, *p* < 0.001; I^2^ = 81.6%), but with no publication bias. In sensitivity analyses, the amount of weight loss, percentage of patients with diabetes and value of the Newcastle-Ottawa Quality Assessment Scale, were all associated with significance of effect. Since age was different in one study, a sensitivity analysis was performed by excluding this study; OR was similar [OR = 0.608 (0.454–0.814), *p* < 0.001]; heterogeneity was reduced but still significant (Q = 35.74, *p* < 0.001, I^2^ = 77.6%) and again no publication bias was detected. **Conclusions**. Bariatric surgery as compared to medical treatment is associated with reduced appearance of incident AF.

## 1. Introduction

Overweight and obesity are associated with higher prevalence of co-morbidities, including cardiovascular diseases (heart failure, ischemic heart disease, stroke), renal failure and cancer, and with reduced life expectancy and reduced quality of life [1]. Recent studies have highlighted the association between obesity and atrial fibrillation (AF), showing that two different kinds of AF (incident AF and persistent/recurrent AF) can occur in obesity. Various studies have shown that obesity is associated with an increased prevalence of incident AF and persistent/recurrent AF [2,3]; in addition, weight gain is associated with increased prevalence of incident AF [4], while no data are available for persistent/recurrent AF. In addition to being a risk factor for the development of well-known comorbidities, such as dyslipidemia, type 2 diabetes mellitus, arterial hypertension and obstructive sleep apnea, obesity may increase the risk of AF through several mechanisms, including structural and electrical remodeling, which contribute to the development of an arrhythmogenic environment. This view is supported by clinical epidemiology [2,3,4], as well as by experimental studies, showing the effect of short-term weight gain in remodeling of heart atria, in increasing fibrous tissue deposition, in increasing expression of endothelin receptors and abnormalities in atrial conduction, in increasing AF inducibility [5], and in increasing pericardial fat volumes; histological samples of the atrial myocardium from regions adjacent to pericardial fat deposits showed infiltration of epicardial fat into the myocardium, resulting in potential voltage abnormalities, conduction block, and increased AF vulnerability [6].

Weight loss, obtained especially through bariatric surgery, can significantly counteract several consequences of obesity [7,8]. Bariatric surgery, as compared with diet, exercise, and drugs, is able to induce sustained and prolonged weight loss, and to prevent dyslipidemia, type 2 diabetes mellitus, arterial hypertension and obstructive sleep apnea, cardiovascular disease, cancer, and kidney disease [9], effects that occur regardless of the patients metabolic status [10]; the effect on the reduction of mortality is debated, as it has been seen that in young patients (age < 43 years), especially in the presence of diabetes it could increase mortality [11]. Bariatric surgery is associated, beyond weight loss, with other important changes, namely improved endothelial function [12] and reduced sub-inflammation [13], reduced prevalence of arterial hypertension [14] left ventricular hypertrophy, improved glucose tolerance and reduced insulin resistance [14], improved quality of life [15], thus possibly representing the ideal treatment in the prevention of AF. It remains to be established if these factors could contribute independently from weight loss in preventing AF.

Pilot studies showed the beneficial effect of bariatric surgery on slowing heart rate [16,17], and heart rate recovery after exercise, which reflects the balance of cardiac autonomic input from the sympathetic and parasympathetic systems. In addition, weight loss, especially sustained weight loss through bariatric surgery, has been shown to be beneficial in reducing progression of incident AF to recurrent AF [18,19]. However, a role for bariatric surgery in prevention of incident AF is still controversial; meta-analyses performed on studies available at the time of publication [20,21], have been contradictory in establishing a role of weight loss in preventing incident AF. The scenario has changed during the last two years since more studies on the effect of bariatric surgery on cardiovascular diseases have been published [22,23]. 

The aim of this study was to understand the role of weight loss in preventing incident AF. Therefore, we performed an updated systematic review and meta-analysis to evaluate the role of prolonged and substantial weight loss through bariatric surgery in the prevention of incident AF in obesity.

## 2. Methods

### 2.1. Search Strategy and Inclusion Criteria

**Figure 1:**
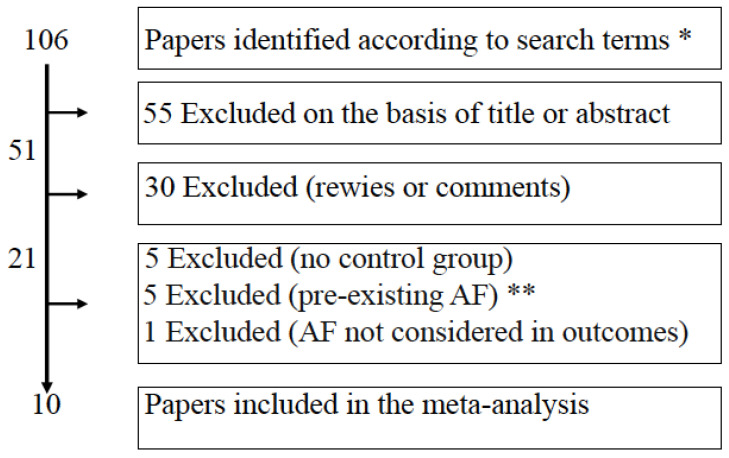
Flow-chart of the analysis performed according to Preferred Reporting Items for Systematic Reviews and Meta-Analyses guidelines (PRISMA). After screening of literature according to search terms, most paper were excluded for reasons indicated in the squares. * Search terms: “atrial fibrillation” AND (“bariatric surgery” OR “biliopancreatic diversion” OR “gastric bypass” OR “roux-en-Y” OR “sleeve gastrectomy” OR “biliointestinal bypass” OR “adjustable gastric banding”) ** AF atrial fibrillation.

This meta-analysis followed the Preferred Reporting Items for Systematic Reviews and Meta-Analyses (PRISMA) guideline [24]. Eligible studies were controlled trials evaluating the appearance of atrial fibrillation in patients undergoing weight loss through bariatric surgery as compared with patients receiving medical treatment. Full reports, published in any language, were included. Two independent authors (AEP, LC) searched relevant literature in databases including PubMed, Embase, and Cochrane Library from inception until 30 October 2022. The following keyword was used for disease: atrial fibrillation. To define exposure, the following keywords were used: obesity, overweight, weight loss, weight reduction, loss of weight, decrease in weight, weight decrease, weight changes, changes in weight, bariatric surgery (laparoscopic adjustable gastric banding, gastric bypass, sleeve gastrectomy, biliopancreatic diversion, biliointestinal bypass). The title and abstracts written in English language were reviewed to recognize eligible studies on the impact of weight loss on the appearance of atrial fibrillation. Additional studies were also manually searched through the references cited in reviews. Cohort as well as case-control studies were included. If the results of one study were reported in more publications, only the most recent and complete data were included in analysis. When required, authors of the studies were also contacted by mail to yield more details. The following studies were excluded: descriptive studies, editorials, review articles, systematic reviews and meta-analyses, and studies that did not provide risk ratios or effect sizes. Decisions on trials to include were taken by the authors (AEP, LC, CLR, SM, ET, FF), and disagreements were resolved by discussion. The reason for exclusion of other trials was specified (lack of details, no controls, pre-existing AF, Figure 1). Ten studies [22,25,26,27,28,29,30,31,32,33] fulfilled the inclusion criteria (Table 1 shows details of the 10 studies included in this meta-analysis). The protocol of the meta-analysis has been registered (Prospero, CRD42022374548).

### 2.2. Data Extraction

The following data were extracted: name of the first author, year of publication, type of study, type of bariatric surgery, number of patients who have undergone surgery and number of control patients. For both groups additional items were extracted: percentage of male and female patients, duration of follow-up, mean age, mean BMI (for the first group before surgery and for control at the start of follow-up), percentage of weight lost during follow-up, percentage of patients diagnosed with type 2 diabetes, with hypertension, with coronary artery disease and with heart failure. The details for each study are shown in Table 1. For the analysis, event rate was extracted per outcome parameter for each group and unadjusted and adjusted HRs with their 95% confidence intervals (CIs) for the association with outcome of interest.

### 2.3. Quality Assessment 

Quality of reports was assessed independently by reviewers according to Newcastle-Ottawa Quality Assessment Scale (NOS) for Cohort Studies [34]. The NOS scale is based on a “star system” in which a study is judged from three broad perspectives: study group selection, group comparability, and ascertaining the outcome of interest. The variables considered are: risk of bias linked to the selection of participants, confounding variables, performance, detection and measurement of exposure, attrition and reporting biases. The length of follow-up was set at a minimum of 5 years to be evaluated as adequate. Disagreement for the quality assessment was resolved by discussion. A score was eventually built, classifying poor, intermediate, or good quality, based on the number of the above criteria available for each paper. The NOS score is reported in Table 2.

### 2.4. Statistical Analysis

Intervention effect (weight loss vs. controls) was expressed as odds ratio (OR), with 95% confidence intervals (CIs); all analyses were performed by a random-effects model according to Hartung and Knapp [35]. Heterogeneity was assessed through Q and I^2^ statistics for each comparison, and potential sources of heterogeneity were discussed where appropriate [36]. Heterogeneity was considered statistically significant for a *p* value < 0.05. Through meta-regression, we also evaluated, the possible role of several patients’ and study characteristics on the incidence of new cases of atrial fibrillation (AF). This was done independently of statistically significant heterogeneity. The dependent variable was the incidence of cases of AF. The role of each covariate in heterogeneity was expressed by Wald test estimated by the meta-regression. The following covariates were included in the meta-regression analysis: number of patients enrolled, age, kind of study (prospective or retrospective), percentage of persons with diabetes mellitus, NOS score, duration of follow-up, body mass index (BMI) of each study (weighted means of intervention and control patients), amount of weight loss and efficacy of treatment (vs. controls) in each study. Meta-regression was performed considering all studies together. In a secondary analysis, we also evaluated the existence of a potential publication bias, that means the tendency of authors and editors to publish studies in which the experimental results achieved statistical significance, more favorably than in studies in which the results were not significant, which would ultimately introduce bias into the overall published literature [37]. Funnel-plot asymmetry was evaluated by using the Egger’s test for small study effects through the meta-bias routine [38]. Sensitivity analyses were also performed by considering the prevalent kind of BS employed in the studies [i.e., restrictive surgery (VGB = vertical banded gastrotomy; AGB = adjustable gastric banding), metabolic surgery (LSG = laparoscopic sleeve gastrectomy; RYGB = roux-en-y gastric bypass), or a mixture of procedures (GA = gastrectomy; GR = gastric resection; GIB = gastric intestinal bypass) plus AGB, LSG and RYGB], percentage of patients with diabetes mellitus, percent weight loss, and exclusion of studies showing substantial variation in basal conditions from other studies. To this end, the Baujat method was used to identify outliers in this model [39]. The Baujat method detects outliers by analyzing the change of the general effect by systematically leaving out one study at a time, i.e., the contribution of this study to the between-study heterogeneity statistic Q, and contribution to I^2^ [40]. A leave-one-out sensitivity analysis was also conducted to assess each study’s influence on the pooled estimate by omitting one study at a time and recalculating the combined estimates for the remaining studies. All statistical analyses were performed by Stata 17 (Stata Corporation, College Station, TX, USA) for MacIntosh.

## 3. Results 

**Figure 2:**
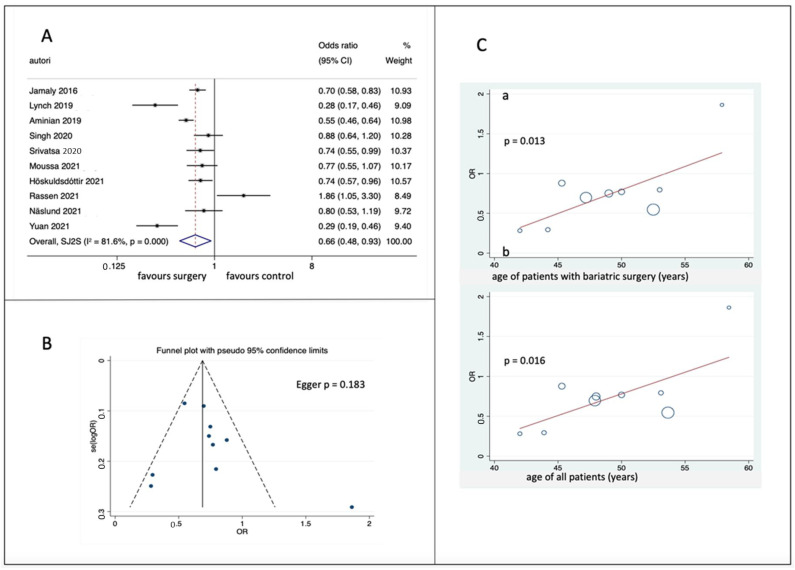
(**A**) Forest plot of pooled hazard ratios of atrial fibrillation [22,25,26,27,28,29,30,31,32,33]; (**B**) funnel plots with 95% CI; (**C**) meta-regression analysis of effect as a function of age of BS patients (a) and of the whole cohorts (b). OR = Odds Ratio; 95% CI = confidence interval; SE, standard error.

Ten studies were included in the meta-analysis (Figure 2A); in 6 out of 10 studies, the effect of bariatric surgery was statistically significant per se, and the overall result was statistically significant [OR = 0.665 (0.475–0.929), *p* = 0.017]. Heterogeneity was significant (Q = 48.98, *p* < 0.001; I^2^ = 81.6%). In contrast, the meta-funnel showed no publication bias (Figure 2B), and at meta-regression, only age correlated with the effect (*p* = 0.013 when considering the age of patients undergoing BS, *p* = 0.016 when considering the age of the whole cohort (Figure 2C). No other item correlated with the OR. Number of patients enrolled, nature of the study (prospective or retrospective), NOS score, duration of follow-up, body mass index (BMI) of each study (weighted means of intervention and control patients), amount of weight lost, percentage of patients with diabetes, did not yield significant correlations at meta-regression. Supplementary File (Supplementary Table S1) reports sensitivity analyses which were performed. 

In a second sensitivity analysis, the effect of the percentage of weight loss was considered; the effect was significant for a weight loss ≥ 22%, [OR = 0.525 (0.316–0.872), 0.013], with lower but still significant heterogeneity (Q = 25.52, *p* < 0.001, I^2^ = 84.2%); the effect was non-significant for a weight loss < 22%, [OR = 0.827 (0.539–1.271), *p* = 0.387] with lower but still significant heterogeneity (Q = 22.30, *p* < 0.001, I^2^ = 82.1%). Then, the role of the percentage of patients with diabetes was considered; for a percentage < 100% (7 studies), the effect was significant [OR = 0.596 (0.406–0.875), *p* = 0.008], with significant heterogeneity (Q = 30.19, *p* < 0.001, I^2^ = 80.1%); for a percentage = 100% (3 studies), the effect was non-significant [OR = 0.880 (0.394–1.969), *p* = 0.756, NS)], but still with significant heterogeneity (Q = 18.43, *p* < 0.001, I^2^ 89.1%). Finally, the effect was evaluated according to the Newcastle-Ottawa Quality Assessment Scale (NOS); the effect was statistically significant [OR = 0.570 (0.365–0.890), *p* = 0.013] for higher NOS score (above mean NOS), and heterogeneity was significant (Q = 29.64 *p* < 0.001, I^2^ = 83.1%); the effect was non-significant for NOS score below mean NOS [OR = 0.840 (0.477–1.478), *p* = 0.545], and heterogeneity was still significant (Q = 19.27 *p* < 0.001, I^2^ = 84.43%). Since one study [32] showed a different age from other studies and contributed to I^2^ heterogeneity for more than 13% [39,40], a sensitivity analysis was performed by excluding this study (Supplementary Figure S1); OR was similar [OR = 0.62 (95% CI 0.51–0.76, *p* < 0.001)]; heterogeneity was reduced but was still significant (Q = 35.70, *p* < 0.001; I^2^ = 77.6%), and again the meta-funnel showed no publication bias. At meta-regression BMI correlated with the effect (*p* = 0.052 when considering BMI of patients undergoing BS, *p* = 0.054 when considering BMI of the whole cohort); more, the OR for weight loss < 22% became significant [OR = 0.696 (0.547–0.885), *p* = 0.003)], with small heterogeneity (Q = 9.71, *p* = 0.021, I^2^ = 69.1%); similarly, the OR for percentage of patients with diabetes was significant [OR = 0.629 (0.443–0.893), *p* = 0.010)], and the heterogeneity was small (Q = 3.92, *p* = 0.048, I^2^ = 74.5%). According to the Newcastle-Ottawa Quality Assessment Scale (NOS), the effect was statistically significant [OR = 0.661 (0.498–0.878), *p* = 0.004] with very small heterogeneity (Q = 5.34, *p* = 0.096, I^2^ = 62.6%). The kind of BS procedure employed was the basis for an additional sensitivity analysis: when restrictive surgeries were employed (VGB and AGB, one study), the OR was 0.697 (0.584–0.832), *p* = 0.001; when metabolic surgeries were employed (LSG and RYGB, 6 studies, the OR was 0.531(0.354–0.797), *p* = 0.002, with small but significant heterogeneity (Q = 26.34, *p* = 0.001, I^2^ = 81.0%); finally, when mixed surgeries were considered (see Table 1 for details, the OR was not-significant [1.033 (0.610–1.750), *p* = 0.904]. with small heterogeneity (Q = 7.10, *p* = 0.029, I^2^ = 71.8%); the effect was not different when one study [32] was excluded [OR = 0.824 (0.642–1.057), *p* = 0.128], and heterogeneity was almost nil (Q = 0.33, *p* = 0.564, I^2^ = 0.0%). 

No publication bias was detected. In summary, a fair part of the heterogeneity was due to one study [28]. Finally, also when other methods of analysis were employed, namely fixed model (RR) instead of random model (OR) or direct analysis of RR as reported by the authors of the studies (ES), the effect was always similarly significant (Supplementary Table S2). Supplementary Table S3 reports the Newcastle-Ottawa Quality Assessment Scale (NOS).

Supplementary Table S4 shows a comparison of baseline clinical conditions of patients undergoing bariatric surgery (BS) and controls in the ten studies included in this meta-analysis. A total of 61,197 patients (22,831 patients undergoing BS and 38,366 control patients receiving medical treatment) was considered; from the Table it appears that the former and the latter patients were well matched as to age, BMI, duration of follow-up, percentage of patients with diabetes, with hypertension, with coronary heart disease, with heart failure; as expected, weight loss was different in BS and in control patients. 

## 4. Discussion

Both incident AF and persistent/recurrent AF can occur in obesity. There are similarities and differences; for instance, obesity is associated with increased prevalence of both incident AF and persistent/recurrent AF [2,3], and weight gain is associated with incident AF [4] while no data are available for persistent/recurrent AF. The reasons why obesity is associated with AF are still largely unknown; obesity is a risk factor for the development of several comorbidities, namely diabetes, arterial hypertension and obstructive sleep apnea, dyslipidemia, that can lead to cardiovascular disease such as coronary heart disease and heart failure [41]; obesity may increase the risk of AF through several mechanisms, including structural and electrical remodeling, which contribute to the development of the arrhythmogenic milieux. This has been shown in experimental studies in sheep [5], in which increased pericardial fat volumes are associated with infiltration of epicardial fat into the myocardium, resulting in potential voltage abnormalities, conduction block, and increased AF vulnerability [6].

It is well established that bariatric surgery induces sustained and prolonged weight loss and thus might represent the ideal treatment to prevent AF through weight loss. Weight loss, especially sustained weight loss through bariatric surgery, can be beneficial in reducing the progress of incident AF to recurrent AF [18,19], but large studies and even meta-analyses failed to establish a role of weight loss in preventing incident AF [20,21].

The present meta-analysis shows that bariatric surgery, compared to medical treatment, is associated with reduced appearance of incident AF, similar to what has been seen for persistent/recurrent AF. The effect was statistically significant in six out of ten studies analyzed, and the overall effect was statistically significant. In addition, the effect was related to the percentage of weight lost and was more evident for cohorts not entirely consisting of patients with diabetes. Lastly, sensitivity analysis showed that after exclusion of one single study [32], the results were even more statistically significant. At meta-regression, only age negatively correlated with effect, and at higher age the effect was lower. After exclusion of one study [32], in which age of the patients was higher than the rest of the studies, and contribution to heterogeneity was significant [39,40], only BMI correlated with effect, indicating a greater effect for higher BMI. All sensitivity analyses reported in the Supplementary File show that the effect was statistically significant under most conditions, and that most of the negative results were due to one single study that was different from others, mainly for the age of the patients. In addition, the effect was similar when calculated as a random model, fixed model, or when evaluated through risk ratios (RR)s as published by the authors of the papers considered in this meta-analysis.

This meta-analysis has several limitations. The first limitation is that, from the studies included, we do not have data on the duration of obesity, even though age of BS and control patients was not different. Secondly, we could only analyze obesity as expressed by body mass index, not through measures of fat distribution [42]. Although BMI is strongly correlated with percent body fat across populations, there are limitations in its predictive ability to estimate body fat for any given individual, in particular in regard of visceral adiposity. In fact, visceral adiposity as measured by WC, WHR, or detailed imaging methods has been shown to be a risk factor independently of BMI [43]. Thirdly, BS is associated with loss of fat-free mass together with fat mass, and muscle loss is an important component of fat free mass [44]. Under conditions of accelerated loss of fat and muscle mass, such as thyrotoxicosis, an increased frequency of accelerated heart rhythm and AF are observed [45]. With BS, no significant changes of thyroid hormones are observed [46], and the only involvement of heart muscle seems to be a remodeling of heart chambers, a situation that does not increase the risk of AF [47]. Finally, different meta-analyses led to different results regarding the effect of BS on incident AF. The main reason is probably the number of studies considered; the strength of our study is that 10 studies were included, while in two previous meta-analyses [20,21] only seven studies were considered. Differently from previous meta-analyses [2,4,18,19,20,21], all analyses were performed by a random-effects model according to Hartung and Knapp [35], that is straightforward and outperforms the standard DerSimonian-Laird method [48], especially when less than ten studies are considered [49]; also, when other methods of analysis were employed, such as fixed model (RR) analysis, or direct analysis of RR as reported by the authors of the studies (ES), the effect was similarly significant; finally, one study [32] was excluded in this meta-analysis because of a clear difference of age of patients compared to other studies. All these differences deserve a comment; first, the role of age might be of importance in AF, as it is in other cardiovascular diseases. In keeping with this possibility, we have shown for the first time that bariatric surgery is associated with reduced all-cause mortality and reduced cardiovascular mortality [7]; however, in a second meta-analysis, we were able to show that age can be of importance in affecting prevention of all-cause mortality and of mortality due to cancer and diabetes [8]. A different possibility is that the amount of weight loss is important, as indicated in this meta-analysis and as reported by Jones et al. [50], who showed that a weight loss of less than 5% is not able to prevent incident AF. The quality of studies assessed through the NOS score might also be of importance, as indicated by our sensitivity analysis; in fact, the effect was statistically significant for higher NOS score (i.e., above mean NOS), but was non-significant for NOS score below mean NOS score.

One should also remember that bariatric surgery does not only mean prolonged and substantial weight loss; bariatric surgery is associated with reduced incidence of diabetes, cardiovascular disease, cancer, and liver and kidney disease [9], effects that occur regardless of the patients’ metabolic status [10].

In summary, bariatric surgery is associated with reduced incident AF in patients affected by obesity. This might be instrumental in reducing cardiovascular morbidity and mortality in people with obesity [41], but more studies are needed to determine the amount of weight loss needed to prevent AF. Moreover, a causal relationship still needs to be confirmed between weight loss and prevention of AF; bariatric surgery is associated, beyond weight loss, with other important changes, namely improved endothelial function [12] and reduced sub-inflammation [13], reduced prevalence of arterial hypertension [14] and of left ventricular hypertrophy, improved glucose tolerance and reduced insulin resistance [14,16], improved quality of life [15]. It remains to be established if these factors, not clearly defined in the studies considered, could also contribute independently from weight loss in preventing AF.

## 5. Conclusions

Bariatric surgery as compared to medical treatment is significantly associated with reduced incident AF, possibly mainly through prolonged and sustained weight loss. The present report is of great value, and consistent with previous data demonstrating that prolonged and sustained weight loss prevent progression of incident AF to recurrent AF.

## Figures and Tables

**Table 1:** Details of the studies analyzed.

Authors and Year	Ref.	Study			Bariatric (BS) Surgical Procedure				BS Patients (% Women)	Follow-Up (y)	Age (y)	BMI (kg/m^2^)	Control Patients (% Women)	Follow-Up (y)	Age (y)		BMI (kg/m^2^)
Jamaly 2016	[25]	cohort prospective			VGB (68%) AGB (19%), RYGB (13%)				2000 (70.7)	19	47.2	42.4	2021 (71.2)	19	48.6		40.1
Lynch 2019	[26]	cohort retrospective			RYGB or LSG				2522 (83.1)	6.2	42	47.1	2522 (83.4)	8	42		47.7
Aminian 2019	[27]	cohort retrospective			RYGB or LSG or AGB or BPD				2135 (70.2)	3.3	52.5	45.1	10734 (68.4)	4	54.8		42.6
Singh 2020	[28]	cohort retrospective			RYGB or LSG or AGB-or BPD				5087 (80.4)	3.9	45.3	--	9858 (81.1)	3.9	45.3		--
Srivatsa 2020	[29]	cohort retrospective			RYGB or LSG				1581 (78.7)	5.5	49	--	3162 (78.7)	5.5	49		--
Moussa 2021	[30]	cohort retrospective			Various BS procedures				3077 (77.3)	12.3	50	43.5	3077 (77.3)	12.3	50		43.1
Höskuldsdóttir 2021	[31]	cohort retrospective			RYGB				5321 (66.6)	4.5	49	42	5321 (68.1)	4.5	47		41
Rassen 2021	[32]	cohort retrospective			44% GA, 8% GR, 50% GIB				291 (69.2)	2.5	57.9	42.6	461 (61.6)	2.5	59		42.1
Näslund 2021	[33]	cohort prospective			RYGB or LSG				509 (42.8)	4.6	53	40.6	509 (42.8)	4.6	53.2		39.7
Yuan 2021	[22]	cohort retrospective			RYGB				308 (82.5)	15	44.2	46.4	701 (76.6)	15	43.6		44.8
**Authors and year**	**Ref.**	**Total patients**	**Main BS type**	**Mean age** **(y)**	**Mean Follow-up (y)**	**Mean BMI (kg/m^2^)**	**WL (%) BS patients**	**WL (%) control patients**	**% DM BS patients**	**% DM control patients**	**%** **HTN** **BS**	**%** **HTN con**	**%** **CAD BS**	**% CAD** **con**	**%** **HF** **BS**	**%** **HF** **con**	**NOS score**
Jamaly 2016	[25]	4021	1	47.9	19	41.2	−20	0	17	13	78.3	63.6	3.0	2.9	3.0	2.9	8
Lynch 2019	[26]	5044	2	42	7.1	47.4	−58	−3.8	29	10	42.5	43.5	--	--	4.9	4.6	7
Aminian 2019	[27]	12869	2	53.6	3.65	43.8	−18	--	100	100	91.5	79.8	9.6	5.5	11.1	12.5	6
Singh 2020	[28]	14945	3	45.3	3.9	--	−20	−0.8	22	20	31.5	29.9	.04	3.3	.8	.8	7
Srivatsa 2020	[29]	4743	2	--	5.5	--	−60	--	23	16	37.4	33.7	2.7	2.9			6
Moussa 2021	[30]	6154	3	50	12.3	43.3	−9	+4.1	22	23	41.4	30.3	11.9	16.9	15.7	14.5	6
Höskuldsdóttir 2021	[31]	10642	2	48	4.5	41.5	−22	−4.8	100	100	47.1	59.8	7.4	5.9	2.7	3.7	6
Rassen 2021	[32]	752	3	58.4	2.5	42.3	−20	--	100	100	65.2	71.7	24.6	24.9	15.9	26.6	4
Näslund 2021	[33]	1018	2	53.1	4.6	40.1	−28	--	41	45	88.9	88.4	12.5	12.9	9.8	10	5
Yuan 2021	[22]	1009	2	43.9	15	45.6	−26.5	−8.25	21	39	44.15	56.0	4.9	5.2	.3	3.7	8

BS = bariatric surgery; VGB = vertical banded gastrotomy; RYGB = gastric bypass; LSG = sleeve gastrectomy; AGB = adjustable gastric banding; GA = gastrectomy; GR = gastric resection; GIB = gastric intestinal bypass. Main BS type: 1 = restrictive surgeries (VGB, AGB); 2 = metabolic surgeries (LSG, RYGB); 3 = various surgeries (LSG, RYGB, AGB, GA, GR, GIB) WL = weight loss; DM = diabetes mellitus; HTN = hypertension; CAD = coronary artery disease; HF = heart failure; NOS = Newcastle Ottawa Scale.

**Table 2:** Newcastle Ottawa Scale of the studies considered in this meta-analysis [34]. The explication of this scale is reported in the Supplementary File.

Studies Author/Pub Year	Selection				Comparability		Outcome			Total Score	Conclusion
	1	2	3	4	1	2	1	2	3		
Jamaly 2016 [25]	1	1	1	1	0	1	1	1	1	8	Good
Lynch 2019 [26]	1	1	1	1	0	1	1	1	0	7	Good
Aminian 2019 [27]	1	1	1	1	0	1	1	0	0	6	Intermediate
Singh 2020 [28]	1	1	1	1	0	1	1	1	0	7	Good
Srivatsa 2020 [29]	1	0	1	1	0	1	1	1	0	6	Intermediate
Moussa 2021 [30]	1	1	1	0	0	1	1	1	0	6	Intermediate
Höskuldsdóttir 2021 [31]	1	0	1	1	1	1	1	0	0	6	Intermediate
Rassen 2021 [32]	1	0	1	0	0	1	1	0	0	4	Poor
Näslund 2021 [33]	1	1	1	0	0	1	1	0	0	5	Poor
Yuan 2021 [22]	1	1	1	1	0	1	1	1	1	8	Good

## Data Availability

Data are available from the corresponding author on the basis of reasonable request.

## Supplementary Materials

The following supporting information can be downloaded at: https://www.mdpi.com/article/10.3390/nu15040940/s1, Supplementary Table S1: sensitivity analyses; Supplementary Table S2: comparison of results obtained with random model (OR), fixed model, and risk ratios published by authors; Supplementary Figure S1: Meta-analysis after exclusion of study [32]; Supplementary Table S3: The Newcastle Ottawa Scale [34]; Supplementary Table S4: Comparison of baseline conditions of patients undergoing bariatric surgery (BS) and controls in the ten studies included in this meta-analysis.
