# Supplementary material for: Effect of Prolonged and Substantial Weight Loss on Incident Atrial Fibrillation: A Systematic Review and Meta-Analysis
Source: Nutrients. 2023 Feb 14;15(4):940. doi: 10.3390/nu15040940 (PMC9964297; doi:10.3390/nu15040940)
Supplement: Supplementary file 1 [file nutrients-15-00940-s001.zip › nutrients-2082159-supplementary.pdf]

Supplementary File to
Effect of prolonged and substantial weight loss on incident atrial fibrillation. A systematic review and meta-analysis
Antonio E. Pontiroli, Lucia Centofanti, Carel Le Roux, Silvia Magnani, Elena Tagliabue, Franco Folli

Supplementary Table S1. sensitivity analyses
Supplementary Table S2. comparison of results obtained with random model (OR), fixed model, and risk ratios published by authors
Supplementary Figure S1. Meta-analysis after exclusion of study [32].
Supplementary Table S3. The Newcastle Ottawa Scale [34]
Supplementary Table S4. Comparison of baseline conditions of patients undergoing bariatric surgery (BS) and controls in the ten studies included in this meta-analysis

Supplementary Table S1. sensitivity analyses

Sensitivity analysis	Studies	OR (95% CI), p	Heterogeneity (Q), p, I2
Bariatric surgery vs controls	10	0.665 (0.475-0.929), 0.017	48.98, p = 0.001, 81.6%
Percent weight loss > 22%	5	0.525 (0.316-0.872), 0.013	25.52, p = 0.001, 84.3%
Percent weight loss < 22%	5	0.827 (0.539-1.271), 0.387	22.30, p = 0.001, 82.1%
Percent weight loss < 22% *	4	0.696 (0.547-0.885), 0.003	9.71, p = 0.021, 69.1%
Percentage of patients with diabetes < 100	7	0.596 (0.406-0.875), 0.008	30.19, p = 0.001, 80.1%
Percentage of patients with diabetes = 100	3	0.880 (0.394-1.969), 0.756	18.43, p = 0.001, 89.1%
Percentage of patients with diabetes = 100 *	2	0.629 (0.443-0.893), 0.010	3.92, p = 0.048, 74.5%
Newcastle Ottawa Scale > mean value	6	0.570 (0.365-0.890), 0.013	29.64 p = 0.001, 83.1%
Newcastle Ottawa Scale < mean value	4	0.840 (0.477-1.478), 0.545	19.27 p = 0.001, 84.4%
Newcastle Ottawa Scale < mean value *	3	0.661 (0.498-0.878), 0.004	5.34 p = 0.096, 62.6%

* excluded study [32]

Supplementary Table S2. comparison of results obtained with random model (OR), fixed model, and risk ratios published by authors

Sensitivity analysis	Studies	OR (95% CI), p	RR (95% CI), p	ES (95% CI, p
Bariatric surgery vs controls	10	0.665 (0.475-0.929), 0.017	0.687 (0.564-0.839), 0.001	0.687 (0.576-0.819), 0.001
Percent weight loss > 22	5	0.525 (0.316-0.872), 0.013	0.508 (0.310-0.832), 0.007	0.735 (0.637-0.849), 0.001
Percent weight loss < 22%	5	0.827 (0.539-1.271), 0.387	0.807 (0.630-1.033), 0.088	0.832 (0.609-1.136), 0.247
Percent weight loss < 22 *	4	0.696 (0.547-0.885), 0.003	0.712 (0.596-0.851), 0.001	0.757 (0.609-0.875), 0.001
Percentage of patients with diabetes < 100	7	0.596 (0.406-0.875), 0.008	0.633 (0.493-0.812), 0.001	0.706 (0.557-0.895), 0.004
Percentage of patients with diabetes = 100	3	0.880 (0.394-1.969), 0.756	0.855 (0.539-1.356), 0.506	0.806 (0.581-1.119), 0.197
Percentage of patients with diabetes = 100 *	2	0.629 (0.443-0.893), 0.010	0.649 (0.505-0.834), 0.001	0.770 (0.619-0.958), 0.019
Newcastle Ottawa Scale > mean value	6	0.570 (0.365-0.890), 0.013	0.610 (0.458-0.812), 0.001	0.647 (0.499- 0.840), 0.001
Newcastle Ottawa Scale < mean value	4	0.840 (0.477-1.478), 0.545	0.821 (0.578-1.166), 0.270	0.759 (0.612-0.942), 0.012
Newcastle Ottawa Scale < mean value *	3	0.661 (0.498-0.878), 0.004	0.670 (0.549-0.819), 0.001	0.810 (0.688- 0.954), 0.012

* excluded study [32]

Supplementary Figure S1. Meta-analysis after exclusion of study [32].
A. Forest plot of pooled hazard ratios of atrial fibrillation; B. funnel plots with 95% CI; C. meta-regression analysis of effect as a function of BMI of BS patients (A) and of the whole cohort (B). OR = Odds Ratio; 95% CI = confidence interval;

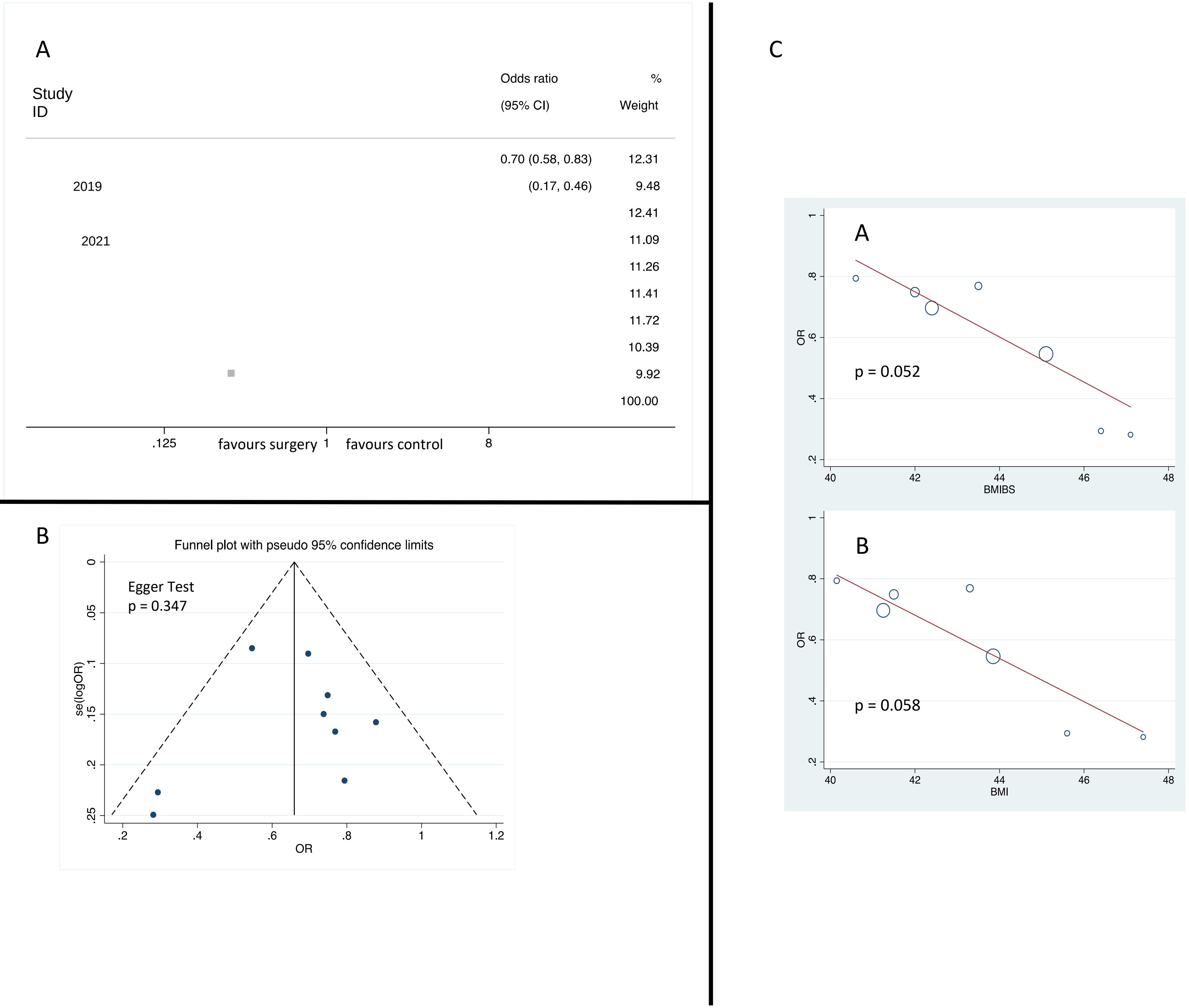

Supplementary Table S3. The Newcastle Ottawa Scale [34]

The Newcastle Ottawa Scale accredits a 1star (=yes, when adequate quality was assessed) or 0 (=no) for specific point in three subcategories.

Selection

1- Representativeness of the exposed cohort
2- Selection of the non-exposed cohort
3- Ascertainment of exposure
4- Demonstration that outcome of interest was not present at start of study

Comparability of cohorts on the basis of the design or analysis controlled for confounders

1- The study controls for age, sex and marital status
2- Study controls for other factors

Outcome

1- Assessment of outcome
2- Was follow-up long enough for outcomes to occur? Minimum of 5 years
3- Adequacy of follow-up of cohorts

According to the total score, the Newcastle-Ottawa scales was then expressed as good, intermediate, or poor quality study:

- Good quality: 3 or 4 stars in selection domain AND 1 or 2 stars in comparability domain AND 2 or 3 stars in outcome/exposure domain
- (total score 7 to 9)
- Fair quality: 2 stars in selection domain AND 1 or 2 stars in comparability domain AND 2 or 3 stars in outcome/exposure domain
- (total score 6)
- Poor quality: 0 or 1 star in selection domain OR 0 stars in comparability domain OR 0 or 1 stars in outcome/exposure domain
- (total score 4 to 5)

Supplementary Table S4. Comparison of baseline conditions of patients undergoing bariatric surgery (BS) and controls in the ten studies included in this meta-analysis.

	BS patients	Control patients	p
Number	22831	38366	
% women	72.1±11.88	70.9±11.95	NS
Age (years)	49.0±4.68	49.2±5.25	NS
BMI (kg/m^2)	43.7±2.28	42.6±2.63	NS
Follow-up (years)	7.7±5.68	7.9±5.59	NS
% diabetes	47.5±36.80	46.6±38.42	NS
% hypertension	56.8±22.31	55.7±20.89	NS
% coronary heart disease	8.5±7.40	8.9±7.69	NS
% heart failure	7.1±6.14	8.8±8.16	NS
% weight loss	28.1±17.04	2.9±3.29	0.001

Absolute numbers and percentages. Mean ± SD.
BMI = body mass index; % = percentage; NS = non-significant
